# Beyond awareness: social media, artificial intelligence, and the PCOS knowledge gap among women in Palestine

**DOI:** 10.3389/fpubh.2026.1893070

**Published:** 2026-07-29

**Authors:** Aisha M. Zoabi, Layan A. Salah, Tasneem Z. Deeb, Jana N. Igbaria, Adele A. Mustafa, Qamar M. AbedRabbo, Sadeen F. Amer, Deema N. Al-Muhtaseb, Taimaa Z. Al-Haddad, Dyalla A. Kayed, Mayar Z. Deeb, Dareen A. Al Qasrawi, Alhareth M. Amro, Salahaldeen Deeb

**Affiliations:** 1 Faculty of Medicine, Al-Quds University, Jerusalem, Palestine; 2 Faculty of Medicine, An-Najah National University, Nablus, Palestine; 3 Faculty of Medicine, Arab American University, Ramallah, Palestine; 4 Faculty of Medicine, Hebron University, Hebron, Palestine; 5 Faculty of Medicine, Palestine Polytechnic University, Hebron, Palestine

**Keywords:** artificial intelligence, awareness, ChatGPT, digital health, health literacy, knowledge, Palestine, PCOS

## Abstract

**Background:**

Polycystic ovary syndrome (PCOS) requires sustained health literacy, yet the degree to which digital exposure translates into accurate understanding remains uncertain in Palestine. This study evaluated PCOS awareness, objective knowledge, social media influence, and artificial intelligence (AI) use among Palestinian women.

**Methods:**

An analytical cross-sectional online survey was conducted among 981 Palestinian women aged 18 years or older between January and February 2026 using convenience and snowball recruitment through digital platforms. The questionnaire assessed sociodemographic characteristics, self-reported physician-diagnosed PCOS, information sources, awareness, factual knowledge, social media influence, credibility behavior, and use of ChatGPT or similar generative conversational AI tools for PCOS-related information. Descriptive, bivariate, correlation, and multivariable models were applied. Because recruitment was online and non-probability based, findings should be interpreted as reflecting respondents in this sample rather than population-level estimates for all Palestinian women.

**Results:**

Participants had high awareness (mean 38.5 ± 7.6 of 50), with 64.8% categorized as highly aware, but objective knowledge was moderate (mean 10.9 ± 2.3 of 20), with only 3.2% achieving good knowledge. Social media was the most common information source (37.2%) and correlated positively with awareness (*r* = 0.35) and knowledge (*r* = 0.23), both *p* < 0.001. AI use was reported by 56.6% and was associated with higher unadjusted awareness and knowledge; however, it was not an independent predictor after adjustment. Medical or health related background, physician diagnosed PCOS, and social media influence independently predicted awareness and knowledge.

**Conclusion:**

Among respondents in this online Palestinian sample, PCOS recognition was broad, but actionable objective knowledge remained incomplete. Social media exposure and AI use were common; however, AI use was not independently associated with awareness or knowledge after adjustment, suggesting that it may reflect broader digital information-seeking rather than independently improving PCOS literacy. Structured, clinician-supported, and culturally responsive digital health literacy programs are needed to address misconceptions and support safer appraisal of online and AI-generated PCOS information.

## Introduction

Polycystic ovary syndrome is among the most common endocrine and metabolic disorders affecting women of reproductive age, with recent global estimates indicating that approximately 10–13 percent of reproductive aged women are affected and that many remain undiagnosed (1, 2). The syndrome is clinically heterogeneous and may involve menstrual irregularity, hyperandrogenism, infertility, insulin resistance, weight related concerns, dermatological manifestations, and long term cardiometabolic risk (3, 4). Because PCOS extends beyond reproduction and often continues across the life course, effective management depends not only on diagnosis but also on a woman’s ability to understand symptoms, recognize complications, evaluate information, and engage in sustained self-care.

Despite the availability of international guidelines, PCOS continues to be associated with delayed diagnosis, inconsistent counseling, and patient dissatisfaction with the information received during clinical encounters (5). Health literacy is therefore central to PCOS care because it reflects the capacity to access, understand, appraise, and apply health information in real decisions (6). In practical terms, women who have heard of PCOS may still misunderstand its diagnostic criteria, underestimate metabolic risk, overestimate infertility, or accept unsafe online claims about supplements and permanent cures. This creates an awareness knowledge gap in which familiarity with the term does not necessarily translate into accurate and clinically useful understanding.

The rapid expansion of digital health information has made this gap more important. Social media platforms provide immediate access to personal stories, educational posts, advertisements, and peer support communities, and they are particularly influential among young adults (7). However, digital exposure is not equivalent to knowledge quality. Users with limited digital health literacy may struggle to assess credibility, separate lived experience from evidence, or recognize commercially motivated content (8). In PCOS, where symptoms are variable and treatment is individualized, inaccurate online messages can reinforce fear, confusion, unnecessary self-treatment, and delayed consultation.

Health misinformation on social media has become a recognized public health concern because platforms often reward engagement, emotion, and repetition rather than accuracy (9, 10). At the same time, generative artificial intelligence tools such as ChatGPT are reshaping how people seek health information by offering rapid, interactive, and personalized responses (11). These tools may clarify medical terminology and help users prepare questions for physicians, yet concerns remain about accuracy, missing context, source transparency, bias, and the risk that users may treat AI output as a substitute for professional care (12).

Previous regional studies have examined general awareness, knowledge, and perceptions of PCOS among women in neighboring Arab populations; however, most have treated digital sources as broad information channels rather than evaluating how social media influence, credibility-checking behavior, and generative conversational artificial intelligence relate to objective PCOS knowledge. Evidence from Palestine remains limited, particularly in relation to how women use digital platforms to understand PCOS in a setting where access to specialized reproductive and endocrine care may be constrained. Therefore, the key research gap is not only whether women have heard of PCOS, but whether exposure to social media and AI-based tools translates into accurate, actionable, and clinically meaningful knowledge. The novelty of this study lies in its simultaneous assessment of PCOS awareness, objective knowledge, social media influence, credibility and risk behavior, and ChatGPT or similar AI use among women in Palestine.

These questions are especially relevant in Palestine, where women navigate PCOS within a health system affected by limited resources, fragmented services, movement restrictions, occupation related barriers, and inequities in access to specialized care (13, 14). In such settings, digital tools may compensate for gaps in access, but they may also magnify misinformation when professional guidance is delayed or unavailable. Accordingly, this study aimed to assess PCOS awareness, objective knowledge, social media influence, credibility behavior, and AI-based information seeking among women in Palestine, and to identify factors associated with awareness, knowledge, and AI use. By linking prior regional PCOS awareness research with emerging digital health behaviors, this study addresses an important gap in understanding whether digital platforms support or merely increase exposure to PCOS-related information.

## Methodology

### Study design and setting

This analytical cross-sectional study was designed to evaluate PCOS awareness, objective knowledge, social media influence, credibility behavior, and artificial intelligence-based information seeking among women in Palestine. Data were collected between January and February 2026 using an anonymous self-administered online questionnaire. The electronic format was selected because the study focused on digital health information behavior and because face-to-face data collection in Palestine may be restricted by fragmented geography, movement barriers, variable transport access, and limited availability of specialized reproductive health services. The survey was circulated through convenience and snowball sampling using social media channels and peer-to-peer sharing to reach respondents from cities, villages, rural areas, and camps. This non-probability recruitment strategy allowed broad digital dissemination but may have preferentially reached younger, educated, medically connected, and digitally active women. Therefore, the findings should be interpreted as describing the recruited online sample rather than providing nationally representative prevalence estimates for all women in Palestine. The cross-sectional design was appropriate for examining associations but cannot establish temporal direction or causality.

### Participants

Eligible respondents were women residing in Palestine who were aged 18 years or older, able to complete the Arabic online questionnaire, and willing to provide electronic informed consent before accessing the survey. Participants were excluded if they were younger than 18 years, did not reside in Palestine, declined consent, submitted an incomplete questionnaire without the core outcome variables, or provided internally inconsistent responses identified during data screening. The survey logic prevented progression without consent and eligibility confirmation. Women from medical and health-related fields were intentionally retained because one objective was to compare awareness and knowledge across educational and professional backgrounds rather than restrict the sample to non-medical respondents. After eligibility screening and data cleaning, 981 women were included in the final analytic sample.

For AI-related analyses, use of generative AI was treated as an exposure variable rather than an inclusion criterion. AI users were defined as respondents who reported using ChatGPT or similar generative conversational AI tools specifically to seek PCOS-related information. Respondents who used social media, websites, search engines, doctors, university sources, or family and friends without reporting ChatGPT or similar AI use were categorized as non-AI users. This definition was used to reduce exposure misclassification and to distinguish generative AI use from general internet or social media use. PCOS status was assessed by asking participants whether they had previously received a physician diagnosis of PCOS. Because this diagnosis was self-reported and was not verified using medical records, laboratory findings, ultrasound reports, or standardized diagnostic criteria, it should be interpreted as self-reported physician-diagnosed PCOS and may be subject to misclassification.

### Questionnaire development and measures

The questionnaire was developed through structured adaptation of previously used PCOS awareness instruments together with investigator-developed items addressing digital health information behavior. The awareness and knowledge components were informed by regionally applied PCOS questionnaires from Jordan and the United Arab Emirates (15, 16). The initial questionnaire was prepared in English, translated into Arabic, and reviewed by independent bilingual experts to ensure linguistic accuracy, semantic equivalence, cultural appropriateness, and clarity for online administration among Palestinian women. The adapted items were reviewed for face and content validity, including relevance to PCOS, clarity of wording, appropriateness for the Palestinian context, and alignment with the study objectives. Items addressing AI use were developed to capture PCOS-specific use of ChatGPT or similar generative conversational AI tools rather than general internet searching.

The AI section assessed whether respondents had used such tools for PCOS-related information, frequency of use, topics searched, perceived usefulness, trust, verification behavior, and perceived influence on health decisions. The final instrument assessed sociodemographic characteristics, residence, education, field of study or work, marital status, income, self-reported physician-diagnosed PCOS, internet access, main information source, awareness, factual knowledge, social media exposure, credibility and risk behavior, AI use, topics searched using AI, and perceived usefulness of ChatGPT or similar tools. Because the questionnaire was adapted and expanded for this study, its composite scores should be interpreted as study-specific measures of awareness, knowledge, and digital information behavior rather than as diagnostic or externally validated clinical scales.

### Scoring and scale reliability

The PCOS Awareness Scale contained 10 Likert-type statements scored from 1 to 5, producing a total score from 10 to 50. Awareness was classified as low from 10 to 23, moderate from 24 to 36, and high from 37 to 50. The PCOS Knowledge Test included 20 true, false, and “I do not know” statements. Each correct answer was assigned 1 point, whereas incorrect and uncertain responses were assigned 0 points, generating a score from 0 to 20. This approach was used to avoid giving credit for uncertain responses and to reduce guessing-related inflation of knowledge scores. Knowledge was classified as poor from 0 to 7, moderate from 8 to 14, and good from 15 to 20. Social media influence was calculated from four Likert-type items with a possible range from 4 to 20. Credibility and risk behavior was calculated from six items with a possible range from 6 to 30. Among AI users, six perceived usefulness and behavioral impact items generated an AI usefulness score from 6 to 30.

Internal consistency was evaluated using Cronbach’s alpha for multi-item Likert-type scales and an internal consistency coefficient for the dichotomously scored knowledge items. A coefficient of 0.70 or higher was considered acceptable for group-level analysis. The internal consistency values were acceptable for the awareness scale, social media influence scale, credibility/risk behavior scale, and AI usefulness scale, with all multi-item scales showing coefficients above 0.78. Exact reliability coefficients should be reported as follows: awareness scale *α* = 0.77, social media influence α = 0.80, credibility/risk behavior α = 0.82, AI usefulness α = 0.76, and knowledge test coefficient = 0.77. Because the knowledge items covered heterogeneous domains of PCOS, item-level responses were also reported to identify specific misconceptions rather than relying only on the total knowledge score.

### Data management and statistical analysis

Survey responses were exported to a spreadsheet, screened for eligibility, checked for coding consistency, and used to construct the prespecified composite scores.

To reduce bias during data preparation, responses were screened before analysis for eligibility, completeness of core variables, coding consistency, implausible values, and internally inconsistent patterns. Ineligible and incomplete responses were excluded before construction of composite scores. No identifying personal data were collected, and no individual response was manually altered after export. Missing data in core outcome variables were not imputed; analyses were conducted using available complete data for the relevant variables. Because recruitment was online and anonymous, residual risks of selection bias, recall bias, social desirability bias, and undetected duplicate participation cannot be fully excluded and were considered when interpreting the findings.

Continuous variables were summarized as means with standard deviations and 95% confidence intervals where appropriate, while categorical variables were reported as frequencies and percentages. Welch independent samples t tests were used for two group comparisons of awareness and knowledge, and one way analysis of variance was used for comparisons across three or more groups. Chi square tests assessed categorical associations, and Cramer V was used to describe effect size. Pearson correlation coefficients evaluated relationships among awareness, knowledge, social media influence, and credibility behavior. Robust ordinary least squares regression models identified independent predictors of awareness and knowledge scores, and robust logistic regression identified predictors of AI use. Covariates were selected *a priori* and included age, residence, education, income, marital status, medical or health related field, physician diagnosed PCOS, internet access, social media influence, credibility behavior, and AI use where relevant.

Before interpreting the multivariable models, multicollinearity among independent variables was assessed using tolerance values and variance inflation factors. Problematic multicollinearity was prespecified as tolerance below 0.20 or variance inflation factor above 5.0. No evidence of problematic multicollinearity was observed, as all predictors remained within the prespecified acceptable diagnostic thresholds. Model fit and explanatory value were interpreted cautiously, particularly for the knowledge model, because a low R^2^ indicates that a substantial proportion of variation in objective knowledge was not explained by the included variables.

Statistical significance was defined as *p* < 0.05.

### Ethical considerations

The study protocol, questionnaire, and electronic informed consent procedure were reviewed and approved by the Research Ethics Committee of Al Quds University. Respondents were informed about the purpose of the study, the voluntary nature of participation, anonymity, and the research use of submitted data before entering the questionnaire. All procedures followed the principles of the Declaration of Helsinki.

## Results

### Sample characteristics

The analytic sample included 981 women residing in Palestine. Mean age was 22.2 ± 6.5 years, range 18–61 years, and 843 respondents, 85.9%, were aged 18–24 years. Participants lived in cities, 534 or 54.4%, villages or rural areas, 415 or 42.3%, and camps, 32 or 3.3%. Most had bachelor or university education, 846 or 86.2%, and 494, 50.4%, were in a medical or health related field. Physician diagnosed PCOS was reported by 292 women, 29.8%, and regular internet access by 920, 93.8%. Social media was the leading main PCOS information source, 365 or 37.2%, followed by doctors, 290 or 29.6%. AI use for PCOS information was reported by 555 respondents, 56.6%, while doctors were most often selected as the single most helpful source, 368 or 37.5% (Table 1).

**Table 1:** Sample demographics and key characteristics.

Variable	Category	*n*	Percent
Age (years)	Mean ± SD (range)	980	Mean = 22.2 SD ± 6.5
Age group	18–24	843	85.9
	25–34	80	8.2
	35–44	29	3.0
	> = 45	28	2.9
Sex	Female	981	100.0
Place of residence	City	534	54.4
	Village/rural	415	42.3
	Camp	32	3.3
Educational level	Secondary or less	129	13.1
	Bachelor/University	846	86.2
	Diploma/Postgraduate	5	0.5
Field of study/work	Medical/health related	494	50.4
	Non-medical	487	49.6
Marital status	Single	836	85.2
	Married	135	13.8
	Other	9	0.9
Monthly income	Low	165	16.8
	Middle	617	62.9
	High	198	20.2
Physician diagnosed PCOS	Yes	292	29.8
	No	689	70.2
Regular internet access	Yes	920	93.8
	No	61	6.2
Main PCOS information source	Social media	365	37.2
	Doctor	290	29.6
	Websites	118	12.0
	Family/friends	111	11.3
	University	97	9.9
AI used for PCOS information	Yes	555	56.6
	No	426	43.4
Most helpful single source	Doctor	368	37.5
	Social media	300	30.6
	ChatGPT/AI	153	15.6
	Websites	72	7.3
	Family/friends	88	9.0

### Scale performance and overall awareness knowledge profile

The mean awareness score was 38.5 ± 7.6 out of 50. Overall, 64.8% of respondents had high awareness, 31.5% had moderate awareness, and 3.7% had low awareness. Objective knowledge was lower in relative terms, with a mean score of 10.9 ± 2.3 out of 20. Most participants had moderate knowledge, 91.1%, while 5.7% had poor knowledge and only 3.2% achieved good knowledge. The social media influence score averaged 13.9 ± 3.1 out of 20, and the credibility or risk behavior score averaged 16.9 ± 4.5 out of 30. These distributions demonstrate a clear awareness knowledge gap.

### Awareness comparisons

**Figure 1:**
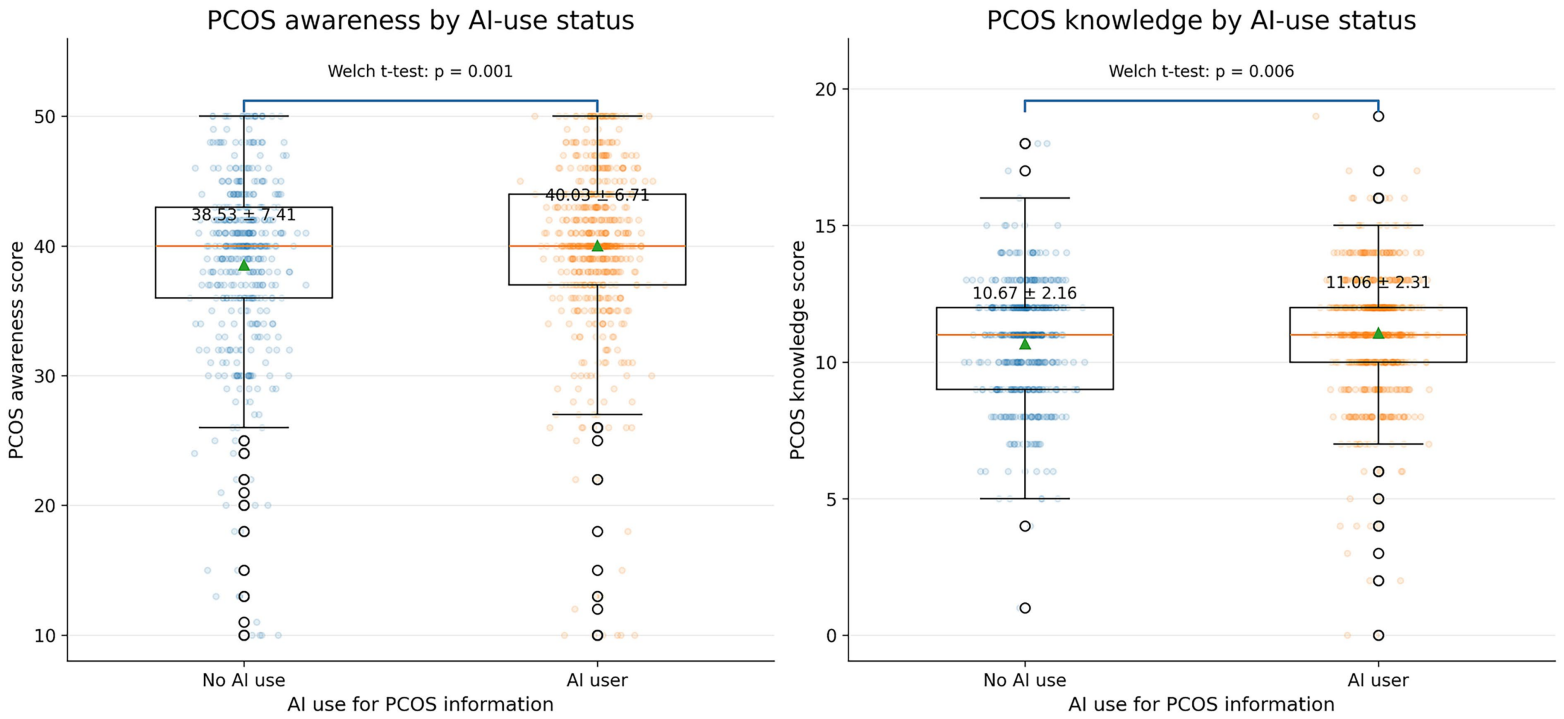
PCOS awareness and knowledge by AI use status. Boxplots with overlaid data points compare awareness and knowledge scores between AI users and non-users, with Welch *t*-test *p*-values shown in the panels.

Awareness differed significantly by age group, *p* < 0.001, with the highest mean among women aged 25–34 years, 41.42 ± 6.44, 95% CI 39.99–42.86, and the lowest among women aged 45 years or older, 33.39 ± 7.35, 95% CI 30.54–36.24. Education was also associated with awareness, *p* = 0.005, whereas residence was not, *p* = 0.278. Medical or health related respondents scored higher than non-medical respondents, 40.54 ± 7.31 versus 36.41 ± 7.35, *p* < 0.001. Physician diagnosed PCOS was associated with higher awareness, 40.87 ± 6.80, 95% CI 40.09–41.65, versus 37.48 ± 7.72, 95% CI 36.90–38.06, *p* < 0.001. By source, university was associated with the highest awareness, 43.69 ± 7.47, and family or friends with the lowest, 35.57 ± 7.87, *p* < 0.001. AI users had higher unadjusted awareness than non-users, 39.43 ± 7.38 versus 37.27 ± 7.75, *p* < 0.001 (Figure 1; Table 2).

**Table 2:** PCOS awareness comparisons across key characteristics.

Variable	Category	*n*	Mean	SD	CI low	CI high	*p*
Age group	18–24	843	38.42	7.62	37.91	38.94	*p* < 0.001
	25–34	80	41.42	6.44	39.99	42.86	
	35–44	29	36.9	7.3	34.12	39.67	
	≥45	28	33.39	7.35	30.54	36.24	
Place of residence	City	534	38.58	7.7	37.92	39.23	*p* = 0.278
	Village/Rural	415	38.54	7.49	37.82	39.27	
	Camp	32	36.38	7.57	33.65	39.1	
Educational level	Secondary or less	129	36.71	6.06	35.65	37.76	*p* = 0.005
	Bachelor/University	846	38.78	7.79	38.25	39.31	
	Diploma/Postgraduate	5	33.2	4.15	28.05	38.35	
Field of study/work	Medical/health related	494	40.54	7.31	39.89	41.18	*p* < 0.001
	Non-medical	487	36.41	7.35	35.76	37.07	
Marital status	Single	836	38.6	7.67	38.08	39.12	*p* = 0.430
	Married	135	37.84	7.22	36.61	39.07	
	Other	9	36.67	7.12	31.19	42.14	
Monthly income	Low	165	36.36	9.22	34.95	37.78	*p* < 0.001
	Middle	617	38.49	7.14	37.93	39.06	
	High	198	40.19	7.14	39.19	41.19	
Physician diagnosed PCOS	Yes	292	40.87	6.8	40.09	41.65	*p* < 0.001
	No	689	37.48	7.72	36.9	38.06	
Regular internet access	Yes	920	38.54	7.65	38.04	39.03	*p* = 0.437
	No	61	37.8	7.04	36	39.61	
Main PCOS information source	Social media	365	36.95	6.65	36.27	37.64	*p* < 0.001
	Doctor	290	39.52	7.42	38.67	40.38	
	Websites	118	39.18	8.16	37.69	40.67	
	Family/friends	111	35.57	7.87	34.09	37.05	
	University	97	43.69	7.47	42.18	45.2	
PCOS content exposure frequency	Never	181	35.54	7.68	34.42	36.67	*p* < 0.001
	Monthly	245	40.07	7.09	39.18	40.96	
	Weekly	448	38.39	7.33	37.71	39.07	
	Daily	107	40.28	8.34	38.68	41.88	
AI used for PCOS information	Yes	555	39.43	7.38	38.82	40.05	*p* < 0.001
	No	426	37.27	7.75	36.53	38	

### Knowledge comparisons

Knowledge differences were statistically significant but smaller. Age group was associated with knowledge, *p* = 0.025, with the lowest mean among women aged 45 years or older, 9.75 ± 3.72, 95% CI 8.31–11.19. Educational level showed a small association, *p* = 0.044, while residence and income were not significant. Medical or health related respondents had higher knowledge than non-medical respondents, 11.14 ± 2.12, 95% CI 10.95–11.33, versus 10.64 ± 2.36, 95% CI 10.43–10.85, *p* < 0.001. Physician diagnosed PCOS was also associated with higher knowledge, 11.26 ± 2.07 versus 10.74 ± 2.31, *p* < 0.001. Knowledge varied by information source, *p* = 0.001, with higher scores among women using websites, 11.32 ± 2.25, and university sources, 11.23 ± 1.74. AI users had slightly higher knowledge than non-users, 11.06 ± 2.31 versus 10.67 ± 2.16, *p* = 0.006 (Table 3).

**Table 3:** PCOS knowledge comparisons across key characteristics.

Variable	Category	*n*	Mean	SD	CI low	CI high	*p*
Age group	18–24	843	10.93	2.22	10.78	11.08	*p* = 0.025
	25–34	80	11.12	1.88	10.71	11.54	
	35–44	29	10.45	2.26	9.59	11.31	
	> = 45	28	9.75	3.72	8.31	11.19	
Place of residence	City	534	10.85	2.18	10.66	11.04	*p* = 0.509
	Village/rural	415	10.92	2.36	10.69	11.15	
	Camp	32	11.31	2.15	10.54	12.09	
Educational level	Secondary or less	129	10.43	2.31	10.03	10.84	*p* = 0.044
	Bachelor/University	846	10.96	2.24	10.81	11.11	
	Diploma/Postgraduate	5	11.2	1.79	8.98	13.42	
Field of study/work	Medical/health related	494	11.14	2.12	10.95	11.33	*p* < 0.001
	Non-medical	487	10.64	2.36	10.43	10.85	
Marital status	Single	836	10.95	2.22	10.8	11.1	*p* = 0.217
	Married	135	10.59	2.36	10.19	10.99	
	Other	9	10.56	3.57	7.81	13.3	
Monthly income	Low	165	10.93	2.21	10.59	11.27	*p* = 0.965
	Middle	617	10.89	2.3	10.71	11.08	
	High	198	10.86	2.15	10.56	11.16	
Physician diagnosed PCOS	Yes	292	11.26	2.07	11.02	11.5	*p* < 0.001
	No	689	10.74	2.31	10.57	10.91	
Regular internet access	Yes	920	10.89	2.26	10.75	11.04	*p* = 0.928
	No	61	10.92	2.13	10.37	11.46	
Main PCOS information source	Social media	365	11	2.2	10.77	11.22	*p* = 0.001
	Doctor	290	10.72	2.22	10.46	10.98	
	Websites	118	11.32	2.25	10.91	11.73	
	Family/friends	111	10.26	2.73	9.75	10.77	
	University	97	11.23	1.74	10.88	11.58	
PCOS content exposure frequency	Never	181	10.23	2.23	9.9	10.56	*p* < 0.001
	Monthly	245	11.22	2.35	10.93	11.52	
	Weekly	448	10.85	2.11	10.65	11.04	
	Daily	107	11.45	2.38	10.99	11.9	
AI used for PCOS information	Yes	555	11.06	2.31	10.87	11.26	*p* = 0.006
	No	426	10.67	2.16	10.47	10.88	

### Correlations and categorical associations

**Figure 2:**
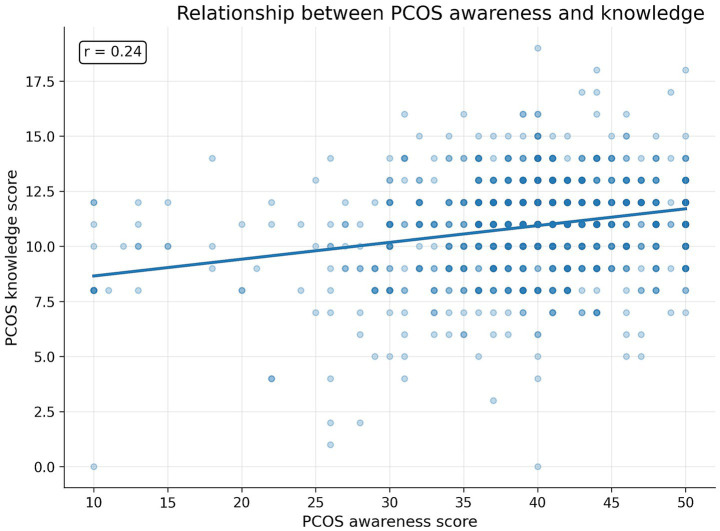
Relationship between PCOS awareness and knowledge. The scatterplot shows the overall positive correlation between awareness and objective knowledge among respondents.

**Figure 3:**
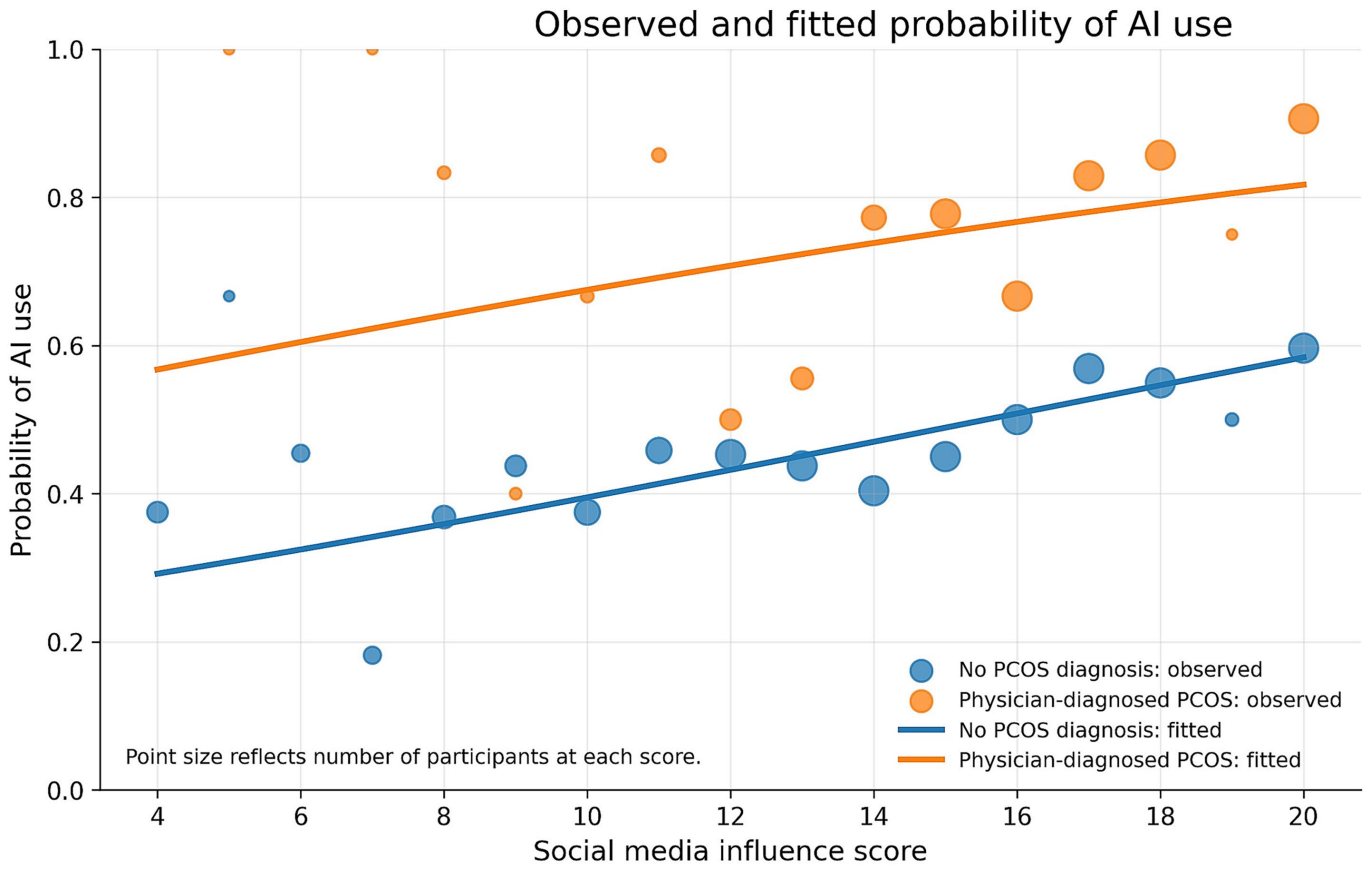
Knowledge versus awareness by AI use status. The scatterplot shows the relationship between PCOS awareness and knowledge scores stratified by AI use for PCOS information, with fitted regression lines and reported Pearson correlations for AI users and non-users.

Awareness and knowledge were positively but modestly related, confirming that awareness did not equal clinically accurate understanding. The overall plotted relationship is shown in Figure 2. When stratified by AI use, the correlation was stronger among non-users, *r* = 0.30, *p* < 0.001, *n* = 426, than among AI users, *r* = 0.17, *p* < 0.001, *n* = 555 (Figure 3). Social media influence correlated with both awareness, *r* = 0.35, *p* < 0.001, and knowledge, *r* = 0.23, *p* < 0.001. Awareness level was associated with field of study or work, chi square = 47.87, *p* < 0.001, Cramer *V* = 0.22, physician diagnosed PCOS, chi square = 40.83, *p* < 0.001, *V* = 0.20, and main information source, chi square = 79.47, *p* < 0.001, *V* = 0.20. Knowledge level showed weaker categorical differentiation. AI use was associated with physician diagnosed PCOS, chi square = 62.91, *p* < 0.001, *V* = 0.25, and most helpful source, chi square = 129.83, *p* < 0.001, *V* = 0.36 (Tables 4, 5).

**Table 4:** Chi square analyses.

Predictor	Awareness level *p*	Knowledge level *p*	AI use *p*
Field of study/work	<0.001	0.074	<0.001
Physician diagnosed PCOS	<0.001	0.084	<0.001
Place of residence	0.444	0.791	
Educational level	<0.001	0.757	
Monthly income	0.002	0.2	
Main PCOS information source	<0.001	0.164	
AI used for PCOS information	<0.001		
Most helpful single source			<0.001

**Table 5:** Multivariable models.

Predictor	Awareness total 95% CI	*p*	Knowledge total 95% CI	*p*	AI use 95% CI	*p*
Age (years)	−0.04 to 0.14	0.233	−0.03 to 0.05	0.538	0.97–1.00	0.955
Medical/health field	3.20–5.00	<0.001	0.14–0.74	0.004	1.00–1.90	0.025
Physician diagnosed PCOS	1.90–3.70	<0.001	0.04–0.66	0.028	2.40–4.70	<0.001
Regular internet access	−2.30 to 0.90	0.393	−0.63 to 0.43	0.707	0.57–1.80	0.997
Social media influence total	0.53–0.89	<0.001	0.11–0.20	<0.001	1.00–1.10	0.002
Credibility/risk total	0.15–0.35	<0.001	−0.04 to 0.03	0.91	1.00–1.10	0.01
AI use yes vs. no	−0.87 to 0.94	0.938	−0.22 to 0.37	0.624		
Awareness total					0.98–1.00	0.945
Knowledge total					0.95–1.10	0.649
Village/rural vs. city	−0.36 to 1.30	0.268	−0.21 to 0.38	0.589	0.86–1.50	0.348
Camp vs. city	−4.10 to 0.17	0.072	−0.26 to 1.20	0.216	0.30–1.40	0.269
Bachelor/university vs. secondary	−1.40 to 0.94	0.678	−0.19 to 0.70	0.257	0.77–1.80	0.475
Diploma/postgraduate vs. secondary	−9.70 to 1.00	0.113	−1.60 to 2.80	0.603	0.17–8.50	0.849
Middle income vs. low	0.87–3.50	0.001	−0.38 to 0.37	0.985	0.52–1.10	0.166
High income vs. low	2.40–5.40	<0.001	−0.44 to 0.44	0.999	0.64–1.60	0.957
Married vs. single	−1.60 to 1.30	0.86	−0.78 to 0.28	0.357	0.27–0.79	0.005
Other marital status vs. single	−5.60 to 3.40	0.641	−2.70 to 2.10	0.83	0.10–2.20	0.335

Models used robust standard errors. Coefficients for awareness and knowledge models are beta coefficients with 95% confidence intervals; coefficients for the AI-use model are odds ratios with 95% confidence intervals. Multicollinearity was assessed using tolerance and variance inflation factor diagnostics, and no problematic multicollinearity was identified based on the prespecified thresholds of tolerance <0.20 or variance inflation factor >5.0. The knowledge model had low explanatory value, *R*^2^ = 0.075, and should therefore be interpreted as identifying limited adjusted associations rather than explaining most variation in objective PCOS knowledge.

### Multivariable models

In the robust awareness model, *R*^2^ = 0.274, indicating that the included variables explained 27.4% of the variation in awareness scores. Factors independently associated with higher awareness were medical or health-related field, beta = 4.1, 95% CI 3.2–5.0, *p* < 0.001, self-reported physician-diagnosed PCOS, beta = 2.8, 95% CI 1.9–3.7, *p* < 0.001, social media influence, beta = 0.71 per point, 95% CI 0.53–0.89, *p* < 0.001, and credibility or risk behavior, beta = 0.25 per point, 95% CI 0.15–0.35, *p* < 0.001. Compared with low income, middle income, beta = 2.2, 95% CI 0.87–3.5, *p* = 0.001, and high income, beta = 3.9, 95% CI 2.4–5.4, *p* < 0.001, were associated with higher awareness. AI use was associated with higher awareness in unadjusted comparisons but was not independently associated with awareness after adjustment, 95% CI −0.87–0.94, *p* = 0.938.

In the knowledge model, *R*^2^ = 0.075, indicating that the included variables explained only 7.5% of the variation in objective knowledge scores. Independent factors associated with higher knowledge were medical or health-related field, beta = 0.44, 95% CI 0.14–0.74, *p* = 0.004, self-reported physician-diagnosed PCOS, beta = 0.35, 95% CI 0.04–0.66, *p* = 0.028, and social media influence, beta = 0.16, 95% CI 0.11–0.20, *p* < 0.001. AI use was associated with slightly higher knowledge in unadjusted analysis but was not independently associated with knowledge after adjustment, 95% CI −0.22–0.37, *p* = 0.624. These findings suggest that AI use may be a marker of broader digital engagement or health information-seeking rather than an independent correlate of objective PCOS knowledge in this model.

**Figure 4:**
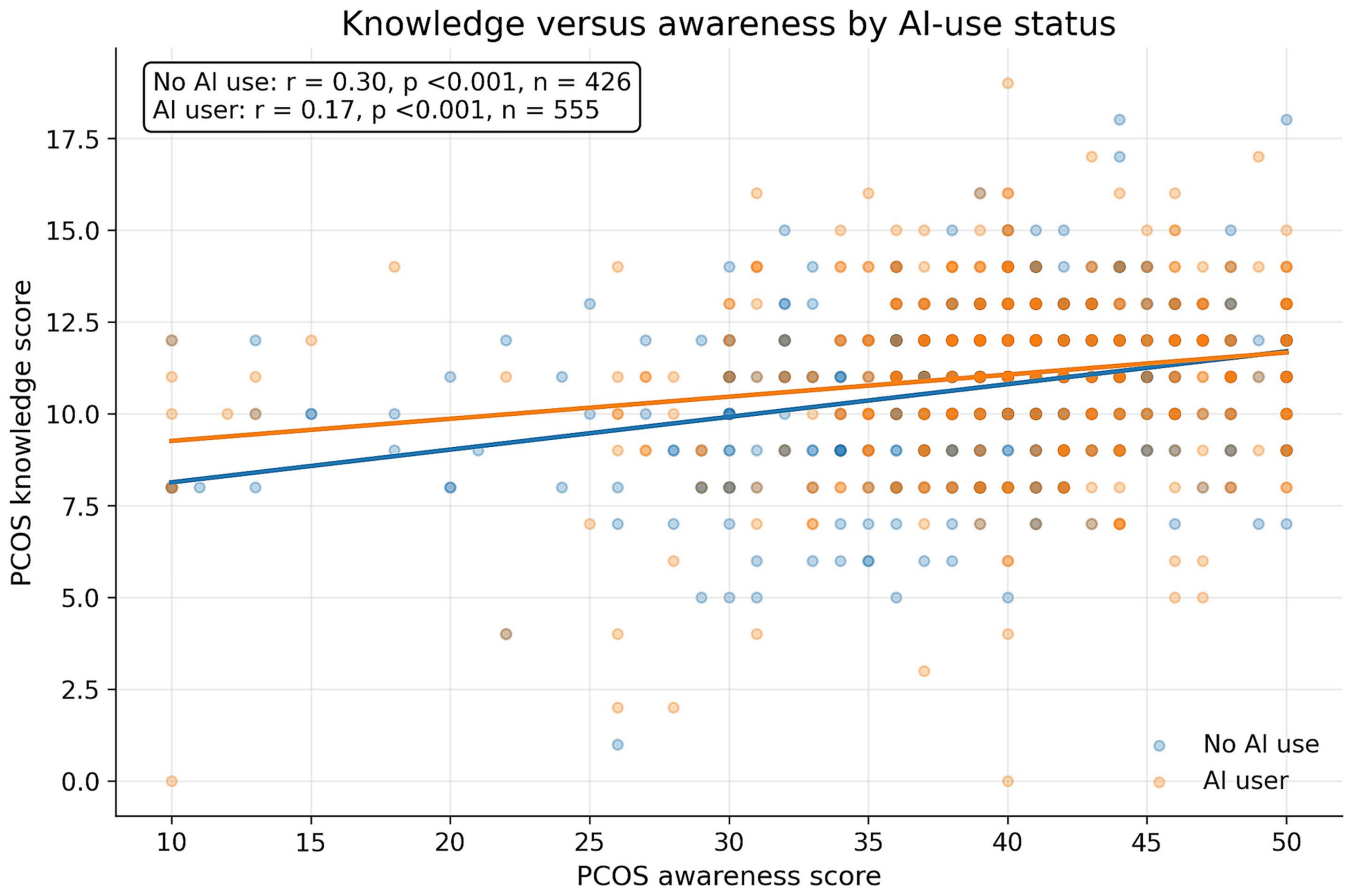
Observed and fitted probability of AI use. Point size reflects the number of participants at each social media influence score, and fitted lines compare participants with and without physician diagnosed PCOS.

In logistic regression, self-reported physician-diagnosed PCOS was strongly associated with AI use, OR = 3.3, 95% CI 2.4–4.7, *p* < 0.001, followed by medical or health-related field, OR = 1.4, 95% CI 1.0–1.9, *p* = 0.025. Married respondents had lower odds of AI use than single respondents, OR = 0.47, 95% CI 0.27–0.79, *p* = 0.005. The fitted probability pattern is displayed in Figure 4 and Tables 4, 5.

### Item level knowledge and AI use patterns

Item level responses showed why moderate total knowledge should be interpreted cautiously. Correct responses were high for irregular menstruation, 90.8%, excess facial or body hair, 89.1%, lifestyle improvement, 87.2%, and importance of early diagnosis, 87.8%. However, only 38.9% correctly rejected diagnosis based only on ovarian cysts, 39.7% recognized increased type 2 diabetes risk, and 35.7% recognized pregnancy complication risk. The lowest correct rates involved contagiousness, 9.5%, infertility inevitability, 18.0%, symptom uniformity, 21.5%, and safety of online promoted supplements, 24.8% (Table 6). Among AI users, use was usually intermittent: 43.6% reported rare use, 28.5% monthly use, 20.4% weekly use, and 5.4% daily use. The most frequent AI search topics were symptoms, 61.4%, diagnosis, 47.9%, and lifestyle, 45.2%. Mean agreement was highest for AI improving understanding and clarifying terms, both 3.64 out of 5, whereas influence on health decisions was lower at 3.09 ± 1.13. The mean AI usefulness score was 19.72 ± 5.25 out of 30 (Table 7).

**Table 6:** Knowledge test items C1–C20.

Item	Question	Correct answer	*n*	Correct %	True %	False %	I do not know %
C1	PCOS is diagnosed only by ovarian cysts on ultrasound.	FALSE	981	38.9	31.3	38.9	29.8
C2	Irregular menstruation is a common feature of PCOS.	TRUE	981	90.8	90.8	5.7	3.5
C3	Excess facial or body hair can be a symptom of PCOS.	TRUE	981	89.1	89.1	9.1	1.8
C4	PCOS can occur in women with normal body weight.	TRUE	981	77.5	77.5	17.5	5
C5	PCOS is associated with insulin resistance.	TRUE	981	57.5	57.5	35.3	7.2
C6	PCOS increases the risk of type 2 diabetes.	TRUE	981	39.7	39.7	53	7.3
C7	PCOS is not linked to cardiovascular risk factors.	FALSE	981	62.6	15.2	62.6	22.2
C8	Lifestyle changes can improve PCOS symptoms.	TRUE	981	87.2	87.2	10.2	2.7
C9	PCOS always causes infertility.	FALSE	981	18	5.8	18	76.1
C10	PCOS may increase pregnancy complications.	TRUE	981	35.7	35.7	51.4	12.9
C11	There is one permanent cure for PCOS.	FALSE	981	37.3	21.7	37.3	41
C12	Medical treatments can help regulate periods in PCOS.	TRUE	981	82.8	82.8	13.7	3.6
C13	PCOS is primarily a hormonal disorder.	TRUE	981	73.3	73.3	22.9	3.8
C14	PCOS symptoms are the same in all women.	FALSE	981	21.5	15	21.5	63.5
C15	Stress and sleep may influence PCOS symptoms.	TRUE	981	77.2	77.2	18.7	4.2
C16	Supplements promoted online are always safe for PCOS.	FALSE	981	24.8	6.2	24.8	69
C17	PCOS can be diagnosed without symptoms.	TRUE	981	44.8	44.8	42.4	12.8
C18	PCOS is contagious.	FALSE	981	9.5	3.6	9.5	87
C19	A woman with regular cycles cannot have PCOS.	FALSE	981	33.6	13	33.6	53.3
C20	Early diagnosis of PCOS is important for long term health.	TRUE	981	87.8	87.8	9.6	2.7

**Table 7:** AI use responses topic selection and AI usefulness items.

Domain	Variable	Category	*n*	Percent	Mean	SD	Range
AI use	F1 Used AI for PCOS information	Yes	555	56.6			
		No	426	43.4			
	F2 Frequency of AI use	Rare	242	43.6			
		Monthly	158	28.5			
		Weekly	113	20.4			
		Daily	30	5.4			
AI topics	F3 Topics searched using AI	Diagnosis	266	47.9			
		Emotional support	136	24.5			
		Fertility	81	14.6			
		Hormones	81	14.6			
		Lifestyle	251	45.2			
		Medications	0	0.0			
		Supplements	4	0.7			
		Symptoms	341	61.4			
		count	1,160	209.0			
AI perceptions	F4 ChatGPT improved my understanding of PCOS	Mean ± SD	543		3.64	0.91	1–5
	F5 ChatGPT clarified medical terms related to PCOS	Mean ± SD	541		3.64	0.94	1–5
	F6 ChatGPT helped me prepare questions for my doctor	Mean ± SD	542		3.23	1.11	1–5
	F7 I trust PCOS information from ChatGPT	Mean ± SD	541		3.16	0.95	1–5
	F8 I verify AI information with medical professionals	Mean ± SD	543		3.46	1.09	1–5
	F9 ChatGPT influenced my health decisions related to PCOS	Mean ± SD	539		3.09	1.13	1–5
	AI usefulness total F4–F9 sum	Mean ± SD	555		19.72	5.25	0–30

## Discussion

This study provides a comprehensive assessment of PCOS awareness, objective knowledge, social media influence, credibility behavior, and AI related health information seeking among women in Palestine. The principal finding is the presence of a distinct awareness knowledge gap. Although nearly two thirds of respondents had high awareness and the mean awareness score was elevated, objective knowledge remained largely moderate and only a small minority reached the good knowledge category. This distinction is important for clinical and public health practice because women may recognize the term PCOS, identify some symptoms, or encounter frequent digital content without understanding diagnostic criteria, metabolic implications, fertility variability, or the limitations of online treatments.

The awareness knowledge gap observed in this study is consistent with evidence from regional PCOS surveys. In Jordan, Abu Tasha and colleagues reported that knowledge and perceptions of PCOS were strongly shaped by prior diagnosis, health related education, and access to credible sources (15). Similarly, a cross sectional study in the United Arab Emirates found that many women had heard of PCOS but that sufficient awareness remained limited and was higher among women with medical exposure or prior diagnosis (16). The present Palestinian findings extend these observations by adding a detailed analysis of social media, credibility behavior, and AI use, demonstrating that digital access does not automatically produce accurate or integrated knowledge.

Several item level findings deserve particular attention. Respondents performed well on visible or commonly discussed manifestations such as menstrual irregularity, hirsutism, lifestyle changes, and the importance of early diagnosis. However, they showed substantial uncertainty about diagnosis by ultrasound alone, diabetes risk, pregnancy complications, symptom variability, the absence of a permanent cure, and supplement safety. These gaps are clinically meaningful because PCOS is a multisystem disorder with reproductive, metabolic, dermatological, and psychological dimensions (17). Incomplete knowledge may delay care seeking, reduce adherence to lifestyle recommendations, increase anxiety about fertility, and expose women to unregulated online products. The results therefore argue for patient education that moves beyond simple symptom recognition toward integrated explanation of mechanisms, risks, and management options.

Medical or health related background and physician diagnosed PCOS were consistent predictors of both awareness and knowledge. These findings are expected but important. Formal education provides structured exposure to evidence based concepts, while diagnosis creates personal relevance and repeated contact with health services. However, the relatively modest knowledge advantage among diagnosed participants suggests that clinical encounters may not always provide enough counseling or reinforcement. Previous international research has shown that delayed diagnosis and inadequate information are closely linked to dissatisfaction among women with PCOS (5). In Palestine, where access to specialist care may be constrained by cost, referral pathways, and mobility barriers, improving the quality of information delivered during each consultation becomes even more essential.

The role of social media in this study was complex. Social media was the most common main information source and social media influence independently predicted both awareness and knowledge after adjustment. This suggests that digital platforms are not purely harmful and may serve as an accessible entry point for women who otherwise have limited contact with reproductive endocrinology services. At the same time, the size of the correlations was modest, and social media exposure cannot be assumed to represent evidence based literacy. Health communication research has repeatedly emphasized that online information can improve reach, peer support, and engagement, while also increasing exposure to inaccurate or commercially driven messages (7, 8). Thus, social media should be understood as a powerful but unregulated learning environment.

The positive association between social media influence and knowledge may also reflect active information seeking rather than direct educational quality. Women who are motivated to learn about PCOS may search more often, follow health pages, compare sources, and ask questions, thereby accumulating more knowledge than passive users. However, without critical appraisal skills, frequent exposure can create overconfidence. The concept of an illusion of explanatory depth explains how individuals may feel they understand complex conditions after repeated superficial exposure even when deeper understanding remains limited (18). This interpretation fits the present results, where overall awareness was high but several clinically important knowledge items showed uncertainty or misconception.

The findings related to AI are particularly relevant because generative conversational tools are becoming part of everyday health information behavior. More than half of respondents reported using AI tools for PCOS information, most commonly for symptoms, diagnosis, and lifestyle. Although AI users had higher awareness and knowledge in unadjusted analyses, AI use was not independently associated with either outcome after adjustment for medical background, self-reported physician-diagnosed PCOS, social media influence, and other covariates. This pattern suggests that AI use may reflect broader digital engagement, motivation to search for health information, or prior exposure to PCOS rather than independently improving PCOS literacy. Because the study did not evaluate the quality, accuracy, source transparency, or clinical appropriateness of AI-generated answers, the findings should not be interpreted as evidence that AI improves PCOS knowledge or health behavior.

This interpretation is supported by the pattern of perceived AI usefulness. Respondents moderately agreed that ChatGPT improved understanding and clarified terminology, while they were less certain that it influenced health decisions. Such a pattern is clinically reassuring to some extent because AI appears to function as a supportive tool rather than a dominant decision maker. Nevertheless, conversational assistants can produce incomplete, inconsistent, or context insensitive health responses, and users may not always verify AI content with clinicians (19, 20). For PCOS, where treatment depends on individual symptoms, reproductive goals, metabolic profile, medication safety, and local availability of care, AI advice must be framed as educational support rather than diagnosis or treatment guidance.

The Palestinian context gives the results additional significance. Women in Palestine encounter health system challenges related to limited resources, fragmented services, movement restrictions, occupation related constraints, and variable access to specialized care (13, 14). These structural pressures can make digital platforms attractive because they are immediate, private, and inexpensive. Yet the same pressures may also increase reliance on unverified information when formal care is delayed or costly. In this context, digital health literacy is not a luxury but a necessary component of reproductive health equity. Interventions should teach women how to identify credible sources, recognize misinformation, verify AI responses, and prepare questions for clinicians.

The results have direct implications for clinical practice. Healthcare providers should not assume that a patient who has heard of PCOS or used social media understands the condition accurately. Short consultations should include targeted checks for common misconceptions, especially regarding ultrasound criteria, infertility, diabetes risk, pregnancy complications, supplements, and the absence of a single permanent cure. Clinicians can ask patients what they have read online or asked AI tools, then correct misunderstandings without blame. This approach can transform digital exposure into a starting point for shared decision making rather than a source of conflict or confusion. Professional societies and medical schools should also support physician led online content that is culturally appropriate, linguistically accessible, and aligned with current guidelines.

From a public health perspective, the study supports shifting from awareness campaigns to knowledge translation programs. Awareness campaigns may successfully introduce the term PCOS, but they are insufficient if women remain unsure about diagnosis, long term risks, and evidence based management. Universities, primary care clinics, women health centers, and community organizations can collaborate to produce structured modules for non-medical students and the wider public. These modules should explain PCOS as a chronic and heterogeneous condition, provide practical guidance on when to seek care, and address common online myths. Because young women were highly represented in the sample, campus based and social media based interventions may be particularly efficient.

Policy makers and health institutions should also consider AI within digital health education frameworks. AI tools are likely to remain widely used, and attempts to discourage use may be unrealistic. A more practical strategy is to teach safe use. Women should be encouraged to ask AI tools for explanations rather than diagnoses, to request sources, to compare answers with trusted medical websites, and to consult clinicians before changing medications or supplements. The broader promise of AI in medicine depends on transparent, human centered implementation that protects patients from bias, overconfidence, privacy risks, and false reassurance (21, 22). In low resource settings, these safeguards are especially important because professional access may be uneven.

This study has several strengths. It included a relatively large sample of Palestinian women, incorporated respondents from different residential settings, and examined not only awareness and knowledge but also the digital pathways through which PCOS information is acquired. The inclusion of social media influence, credibility behavior, and AI use provides a contemporary view of PCOS health literacy in a setting where digital sources may be especially important. The use of multivariable models helped distinguish crude associations from adjusted associations. However, several limitations should be acknowledged. First, the cross-sectional design prevents causal inference and does not allow determination of whether digital exposure preceded or improved awareness or knowledge. Second, online convenience and snowball sampling may overrepresent younger, educated, medically connected, and digitally active women, limiting generalizability to all Palestinian women. Third, the high proportion of medical or health-related respondents may have inflated awareness and knowledge estimates. Fourth, PCOS diagnosis was self-reported as physician-diagnosed and was not verified using clinical records or standardized diagnostic criteria, which may have introduced misclassification. Fifth, information sources, AI use, and credibility behavior were self-reported and may be affected by recall and social desirability bias. Finally, the study did not assess the quality of social media content, the accuracy of AI-generated responses, the quality of user prompts, actual verification practices, clinical outcomes, or real behavior such as consultation, medication use, supplement use, or adherence to medical advice. The low R^2^ of the knowledge model also indicates that most variation in objective knowledge remained unexplained by the measured variables, suggesting that unmeasured factors such as prior counseling quality, health literacy, personal experience, curriculum exposure, and specific online content may be important.

Future research should use representative sampling and mixed methods designs to explore how women interpret PCOS content encountered online and how digital advice affects real health behavior. Qualitative interviews could clarify why women trust specific sources, how they use AI outputs, and what prevents them from seeking professional care. Longitudinal studies could examine whether digital health literacy interventions improve timely diagnosis, consultation behavior, metabolic screening, and treatment adherence. Interventional work is especially needed in Palestine to develop culturally sensitive, low cost, and scalable models that can operate despite structural barriers to access.

## Conclusion

Among respondents in this online Palestinian sample, PCOS awareness was common but did not consistently translate into accurate, integrated, and actionable knowledge. Social media and AI tools were frequently used for PCOS information, particularly among young and digitally connected respondents, but AI use was not independently associated with awareness or knowledge after adjustment. Medical or health-related background, self-reported physician-diagnosed PCOS, and social media influence were the most consistent factors associated with awareness and knowledge. Given the non-probability online sampling, self-reported diagnosis, and cross-sectional design, these findings should be interpreted cautiously and should not be generalized as population-level estimates or causal effects. Future education should move beyond naming PCOS toward explaining diagnostic criteria, metabolic risk, fertility variability, treatment options, and safe appraisal of online and AI-generated content. Clinicians, universities, and public health institutions should collaborate to provide reliable, culturally responsive, and accessible PCOS education for women in Palestine.

## Data Availability

The raw data supporting the conclusions of this article will be made available by the authors, without undue reservation.
